# Automatic Visual Inspection for Industrial Application

**DOI:** 10.3390/jimaging11100350

**Published:** 2025-10-08

**Authors:** António Gouveia Ribeiro, Luís Vilaça, Carlos Costa, Tiago Soares da Costa, Pedro Miguel Carvalho

**Affiliations:** 1 INESC TEC—Institute for Systems and Computer Engineering, Technology and Science, 4200-465 Porto, Portugal; antonio.g.ribeiro@inesctec.pt (A.G.R.); luis.m.salgado@inesctec.pt (L.V.); tiago.a.costa@inesctec.pt (T.S.d.C.); 2 Faculty of Engineering, University of Porto (FEUP), 4200-465 Porto, Portugal; 3 Neutroplast Company, 2590-057 Sobral de Monte Agraço, Portugal; carloscosta@neutroplast.com; 4 Escola Superior de Ciência e Tecnologia, Instituto Superior Politécnico Gaya, 4400-103 Vila Nova de Gaia, Portugal; 5 ISEP, Polytechnic of Porto, 4249-015 Porto, Portugal

**Keywords:** quality control, industrial environment, incremental learning, machine learning, multi-view, defect detections

## Abstract

Quality control represents a critical function in industrial environments, ensuring that manufactured products meet strict standards and remain free from defects. In highly regulated sectors such as the pharmaceutical industry, traditional manual inspection methods remain widely used. However, these are time-consuming and prone to human error, and they lack the reliability required for large-scale operations, highlighting the urgent need for automated solutions. This is crucial for industrial applications, where environments evolve and new defect types can arise unpredictably. This work proposes an automated visual defect detection system specifically designed for pharmaceutical bottles, with potential applicability in other manufacturing domains. Various methods were integrated to create robust tools capable of real-world deployment. A key strategy is the use of incremental learning, which enables machine learning models to incorporate new, unseen data without full retraining, thus enabling adaptation to new defects as they appear, allowing models to handle rare cases while maintaining stability and performance. The proposed solution incorporates a multi-view inspection setup to capture images from multiple angles, enhancing accuracy and robustness. Evaluations in real-world industrial conditions demonstrated high defect detection rates, confirming the effectiveness of the proposed approach.

## 1. Introduction

Product inspection serves a crucial role in ensuring that industrial products are safe, high-quality, and adhere to contemporary standards, subsequently promoting consumer satisfaction and business success. Identifying and correcting flaws early in the production process can reduce the costs of potential recalls, reduce legal risks, and develop a favorable brand reputation [1]. As the role of visual quality control becomes increasingly important along with expected technological development, defect detection, accuracy monitoring, and consistency are deemed critical in production lines in industrial environments.

Quality control practices often rely on traditional methods such as manual inspection, which are most often time-consuming and prone to human error (e.g., manually inspecting bottles one by one). In some scenarios, human labor is still considered a prerequisite: employees carry out tedious manual inspections of manufactured goods to determine and identify potential defects [2]. Despite these limitations, human inspection still plays an important role in industrial environments, particularly in sectors where quality, precision, and compliance with strict standards are critical, since the shipment of nonconforming products can result in significant financial losses and damage to market reputation.

With the introduction of Artificial Intelligence (AI), Computer Vision (CV), and Machine Learning (ML) technologies, significant advancements in object detection in the real world have become widely available. In industrial environments, these technological advancements have enabled a vast improvement in defect detection across various industries [3]. AI models have been implemented in the automotive industry, allowing manufacturers to accurately determine the existence of car defects (e.g., scratches, dents) in mass production runs through the use of visual intelligence systems [4]. Such systems help to reduce the work required from quality control employees, as well as increase efficiency in detecting any form of defects.

This article addresses visual inspection in industrial environments, with a special focus on pharmaceutical bottles. The work was developed in close collaboration with a stakeholder of the sector—Neutroplast [5,6]—to address real-world problems and needs, have available real products, and validate the results. A visual inspection pipeline, capable of integration into production lines, was implemented through the combination of state-of-the-art (SOA) technologies and new developments. The proposed automatic visual inspection module targets regions of interest in the product, i.e., regions where defects are present, signaling when this occurs. This core module incorporates advanced AI-based models and explores an incremental learning strategy to improve flexibility. The core objective is to enable the system to learn and adapt to new types of defects while mitigating catastrophic forgetting through incremental learning methods, particularly Learn without Forgetting (LwF) [7].

Due to a lack of representative data, a representative dataset was developed, incorporating images from both controlled and real-world production settings. These datasets provided a broad range of defects and were used to train and validate the proposed models. The primary goal of this research work was to improve the efficiency and flexibility of defect detection within an industrial environment through an AI-based visual inspection system, reducing the dependence on manual labor. Beyond pharmaceutical bottles, the system was designed to enhance product quality in other industrial applications that present similar defect types. The major contributions from this research work are as follows:

1. Development of a dataset, which includes ground truth data from defects in pharmaceutical bottles, gathered in controlled and real-world industrial environments (Dataset can be made available upon request), to enable assessment in real conditions.
2. Set of baseline experiments to monitor how diverse object detection models cope and react under various conditions presented by the proposed datasets, providing insights into their performance in a real industrial scenario.
3. Implementation of a visual defect detection model and training strategies based on incremental learning through Learn without Forgetting (LwF), capable of addressing the real requirements of a production line.

This research work contains the following sections: Section 2 presents the modern literature consisting of Region of Interest (ROI) detection, visual inspection for defects, defect detection, incremental learning, and relevant datasets. Section 3 presents the datasets chosen as well as a detailed description. Section 4 presents the proposed incremental learning, the implementation strategy, and the performance evaluation metrics. In Section 5, the experimental results are discussed in detail. Lastly, Section 6 concludes this research work.

## 2. Literature Review

### 2.1. Region-of-Interest Object Detection

Region-of-Interest (ROI) object detection methods are commonly applied when it is necessary to isolate the most relevant regions within an image. Within visual inspection in industrial environments, the importance of an automatic ROI selection is crucial, as it ensures that only the most relevant parts of an image are processed. By focusing computational efforts solely on the relevant regions of input images, processing speed can be increased and surrounding noise minimized [8].

In [9], the authors proposed an automated ROI localization and classification system for civil infrastructure assessment using structure-from-motion, Convolutional Neural Networks (CNN), and image registration. This system was validated on 5321 images under diverse conditions. Their approach efficiently extracted relevant ROI and proved to be less demanding and more effective than previous methods.

Study [10] implemented ROI-based methods for identifying marine mammals by filtering irrelevant regions and focusing on the animals. Three approaches were compared: Haar-cascade detection, pixel-wise classification, and CNNs with transfer learning. The CNN-based method was considered to be the most effective, enabling faster and more accurate detection than human analysis.

Article [11] proposed an ROI-based image processing technique for dimensional measurement and defect detection of bearing rings. The system combined optical control, mechanical transmission, image acquisition, and detection software. Performance evaluation demonstrated 97% accuracy with low false detection rates for both good and defective products.

In [12], the authors developed an eleven-layer CNN with visual attention to automate ROI identification for facial expression recognition. Using multiple facial datasets, the method improved both accuracy and visualization quality compared to baseline models.

Study [13] proposed a visual attention model for automatic ROI detection in video sequences, addressing challenges in infrared and dynamic scenes. The bottom-up implicit attention mechanism achieved robust and real-time performance, validated on sea surface video sequences.

### 2.2. Defect Detection

The growing need for quality control in industrial settings has driven advances in defect detection, as traditional human inspection struggles with complex conditions and limited annotated data. To address these challenges, ML and CV techniques, including CNNs, Deep Learning (DL), and object detection algorithms, have been increasingly applied. In paper [14], a survey of CV techniques for detecting defects in industrial settings was conducted, covering image data processing techniques, like image filtering and segmentation, pixel transformation, and image labeling, as well as popular object detection models such as Region-based Convolutional Neural Network (R-CNN) and YOLO. The authors discussed that Faster R-CNN outperforms its predecessors while delivering adequate real-time defect detection, and real experiments using YOLOv3 on automotive wheels and fabrics led to relevant performance results.

The use of DL techniques to detect industrial surface defects offers significant advantages depending on data and resources, as discussed in [15], which addresses DL-based approaches using existing models such as CNN. The work analyzes applications to steel, rail, magnetic tile, and solar cell inspections, implementing various methods such as deep CNN with saliency maps, multilevel-feature fusion, Generative Adversarial Network (GAN) with CNN, MCuePush U-Net, YOLOv3, MobileNetV2, and Faster R-CNN; for example, an improved Faster R-CNN with multi-layer feature integration and Guided Anchoring RPN (GA-RPN) achieved an mAP of 94.62%, 11.26% higher than the original model, but the DL techniques struggled to detect smaller defects. Another DL method for detecting surface defects in industrial settings is ConvNeXt-SegDec-FRB [16], which addresses challenges of limited data and small defect sizes. The two-stage network uses a segmentation network with a ConvNeXt backbone followed by a classification network with a feature retention block to preserve multi-layer semantic features, achieving 93.9% mean accuracy on the DAGM dataset and 85.63% on a private bottle cap defect dataset, confirming the effectiveness of the ConvNeXt backbone and feature retention block module, but requires training on the full dataset.

In [17], a segmentation-based method for surface crack detection in industrial settings is presented. The two-stage architecture includes an 11-layer convolutional segmentation network for pixel-wise defect localization and a decision network for binary image classification, achieving 99.9% average precision on the Kolektor dataset and outperforming commercial software and standard segmentation networks like DeepLabv3+ and U-Net AP, with an inference time of 110 ms. However, this method was designed primarily for segmentation-based surface defects and was tested on a small dataset of 399 images, limiting its ability to generalize to larger, noisier, and more diverse industrial datasets.

A novel CNN-based architecture was presented in [3], proposing a two-stage cascaded autoencoder architecture that combines detection and classification modules. The detection module segments defects using two levels of autoencoder networks, and the classification module categorizes the segmented regions with a compact CNN, achieving 89.60% Intersection-of-Union (IoU) for detection and an accuracy of 86.82% for classification, outperforming thresholding, fully convolutional networks, and HOG-based methods.

Recent advancements in Internet of Things (IoT) infrastructures and DL techniques have increased the adoption of visual inspection for automated quality control in manufacturing lines, enabling accurate detection of defects such as color variations and abnormal patterns. A visual sensing system for precise quality inspection, formally identified as BubCam, is discussed in [18]. It employs a DL-based image segmentation model to inspect ink bags in factories while addressing challenges like light reflection, motion blur, and labeling difficulties. BubCam features multi-camera capture, image processing, segmentation, result fusion, and configuration adaptation, achieving a 34% improvement in accuracy and a 300× reduction in latency compared to manual inspection, even under varying lighting conditions.

Survey [19] explores visual inspection techniques for metallic, ceramic, and textile surfaces, categorizing defects into visible and tactile types and analyzing statistical, structural, filter-based, model-based, supervised, and unsupervised approaches. Experiments showed that statistical and filter-based methods can achieve high detection rates (up to 90%), while the Gabor Filter and Gray Level Co-occurrence Matrix are computationally inefficient, and Neural Networks (NN) methods require large datasets for adequate training. In [20], deep CNN methods for visual defect detection in industrial quality control are explored, covering approaches from manual inspection to traditional and modern automatic optical inspection systems using CNN. It highlights that CNNs improve accuracy, robustness, and real-time inspection capabilities, while also noting challenges such as the need for large labeled datasets, potential generalization issues, architectural complexity, and high computational requirements.

The work in [21] reviews visual inspection methods for detecting and classifying steel product defects, ranging from segmentation, statistical, and spectral approaches to ML and DL. It highlights that the Gabor filter is suitable for small datasets due to its speed and low cost, while YOLO and CNN are more appropriate for larger datasets.

The study in [4] introduces a lightweight DL-based online system for assessing the appearance quality of painted automotive body panels. The system combines multi-viewpoint vision inspection, which mitigates uneven illumination and complex surface structures, with an appearance quality evaluation using TinyDefectRNet, a RetinaNet-based network. Experimental results showed that the proposed system outperformed traditional image processing and ML methods, achieving a top mAP of 87.2% in defect detection.

### 2.3. Incremental Learning

Incremental learning can be characterized as an ML-based approach where a model is capable of continuously retrieving new data and building upon existing knowledge, splitting the overall data into smaller portions to better comprehend each part and extract relevant features. Nevertheless, this process can lead to catastrophic forgetting, when a neural network abruptly loses previously learned knowledge upon learning new tasks, failing to retain past information. To address this, the approach presented in [7] focuses on Learning without Forgetting (LwF). LwF provides a framework for preserving classification performance on previously learned tasks while simultaneously acquiring new ones. It extends the Multiclass Cross-Entropy loss function by excluding indices related to old tasks, thereby incorporating a feedback mechanism from the prior model through knowledge distillation. This approach enables a computationally efficient strategy for maintaining performance on concurrent training tasks without requiring access to the data from earlier tasks.

Study [22] addresses catastrophic forgetting in NN using Elastic Weight Consolidation (EWC), which limits changes to weights that are considered relevant for previous tasks through a quadratic penalty. Experiments showed that EWC outperforms SGD and L2 in supervised learning by retaining prior knowledge while learning new tasks, though efficiency in continuous learning could be improved with better parameter uncertainty estimation, potentially via Bayesian NN. Article [23] explored the combination of YOLO with incremental learning using EWC, showing that introducing new data with EWC helped to preserve accuracy on previously learned categories. Experiments on VOC2012 demonstrated that YOLOv5 with EWC was capable of outperforming YOLO in retaining older knowledge, while label conflicts between old and new data were identified as a source of catastrophic forgetting.

The work in [24] discusses Real-time Incremental Learning for Object Detection (RILOD), which relies on the use of a static reproduction of the original detection network and a dual-network system to introduce new classes without disrupting existing knowledge. Experiments with Fast R-CNN and a ResNet-50 backbone on Pascal VOC 2007 and COCO datasets showed that RILOD effectively avoided catastrophic forgetting for old classes, though new class performance was slightly lower than the baseline.

The study in [25] proposed an RILOD approach using a one-stage object detector with a novel knowledge distillation loss, incorporating samples from previous classes. Experiments on Pascal VOC 2007 (RetinaNet with ResNet-50) and iKitchen (RetinaNet with ResNet-18) showed that the method could learn new classes without forgetting old ones, and using 10 samples per old class improved mAP by 15–40% with a 38x speed-up while only reducing accuracy by 4% compared to using all data. Study [26] addresses catastrophic forgetting in one-stage detectors like YOLO by adapting LwF with prediction-wise weighted cross-entropy and classification loss to handle noisy teacher outputs. Experiments on YOLOv8n using PASCAL VOC 2017 and COCO datasets showed that YOLOv8n with LwF outperformed baseline approaches while effectively mitigating catastrophic forgetting.

### 2.4. Related Datasets

In industrial environments, high-quality datasets are crucial for developing models that operate in complex, real-world conditions, such as defect detection; however, these datasets are often scarce. This section aims to provide an overview of relevant industrial datasets, highlighting their characteristics and applications. Study [17] focuses on developing and evaluating an ML method for automated surface-anomaly detection, specifically cracks on industrial semi-finished electrical commutators. Due to the lack of public datasets, the authors developed the Kolektor Surface Defect Dataset, capturing real-world scenarios with high-quality images (1408 × 512 pixels) of each commutator in eight non-overlapping views, serving as a benchmark for segmentation-based DL approaches.

Study [27] highlights the need for real-world data to develop reliable ML models for surface inspection, particularly for ball screw drives. However, due to the lack of public datasets, the authors had to collect their own data for model development. The authors created a real-world ball screw drives dataset with 21,853 RGB images (150 × 150 pixels), evenly split between defective and non-defective surfaces, capturing challenging examples with small and large inter-class variance to represent the full spectrum of ball screw drive conditions.

Article [28] presents the MVTec anomaly detection dataset to address the lack of real-world industrial datasets for unsupervised anomaly detection. It contains 5354 high-resolution images across 15 categories, including defect-free images for training and over 70 defect types for testing, accompanied by 1888 ground-truth anomaly annotations, with images cropped between 700 × 700 and 1024 × 1024 pixels.

The work in [29] introduces an automated, image-based inspection system targeting surface defects in manufacturing parts, with a focus on mitigating limited and imbalanced datasets. The authors created a dataset from engine blades sourced from real production processes, annotated by experts. The dataset contains 480 high-resolution images (1000 × 1000 pixels) from 20 blades, including 204 healthy surfaces, 149 with scratches, and 48 with nicks, later resized to 224 × 224 pixels for model training.

Study [30] addresses surface defect detection in magnetic tile manufacturing, focusing on building an automated, real-time, and accurate vision-based system. To this end, the authors created a dataset of 1344 images with a cropped ROI of the magnetic tile surfaces. The dataset was divided into six categories: blowhole, crack, fray, break, uneven (grinding uneven), and free (no defects). Image resolutions varied with the ROI, ranging from 300 to 1200 pixels.

## 3. Data Acquisition and Preparation

This section describes the selected datasets and the processes used for the acquisition and preparation of a new one.

### 3.1. Metal Surface Defect Dataset

**Figure 1:**
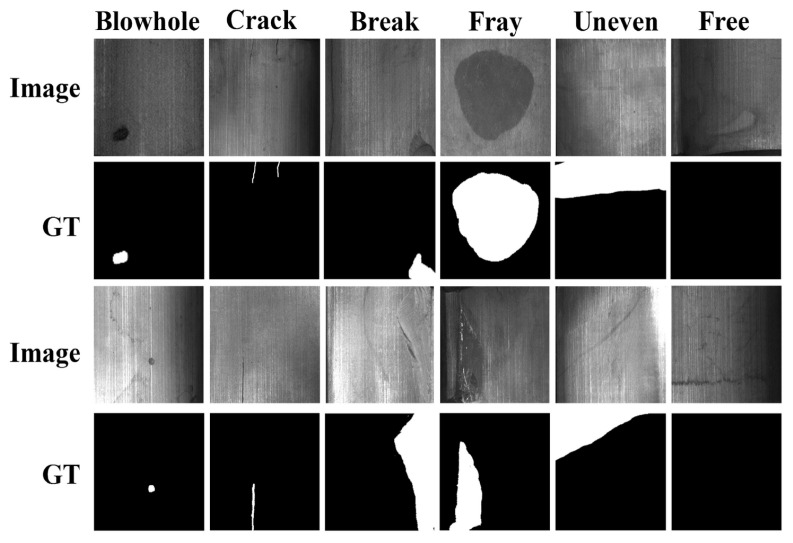
Examples of magnetic tile surface defects, labeled with pixel-level ground truths (GTs) (extracted from [30]).

The Metal Surface Defect Dataset (MSDD) dataset was retrieved from [30]. It was specifically introduced to evaluate model performance under unbalanced data distributions, where some defect classes contain a larger number of samples than others. The dataset contains 2687 high-resolution images (up to 1200 pixels) across six categories: blowhole, break, crack, fray, uneven, and free (non-defect). It includes both original images in JPG format and their segmented ground-truth masks in PNG format. Its directory structure is organized according to defect type, with examples shown in Figure 1. The dataset is organized into six defect-specific directories containing 2687 high-resolution images, which include 115 images of blowholes, 85 of breaks, 57 of cracks, 32 of frays, 952 of non-defects, and 103 of uneven defects.

### 3.2. Blade Surface Defect Dataset

**Figure 2:**
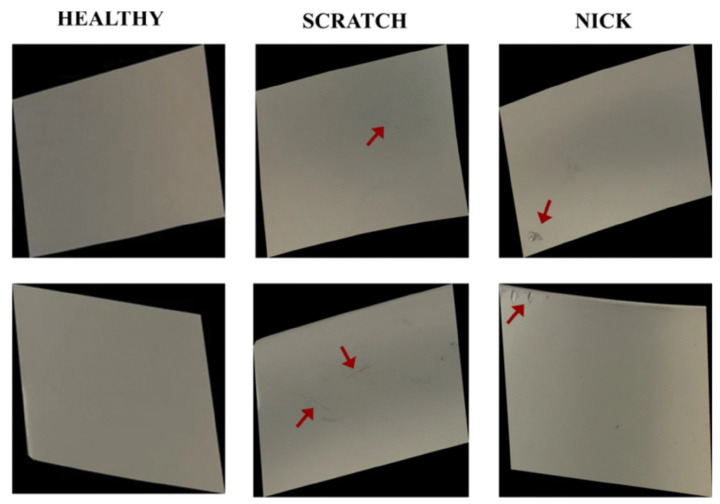
Sample images for each class from the Blade Surface Defect Dataset, where the red arrows indicate the defects present in each image (extracted from [29]).

The Blade Surface Defect Dataset (BSDD) was collected from [29] and is used to evaluate model performance under limited data conditions, in contrast to MSDD, which contains a larger number of samples. It consists of 480 high-resolution images acquired from 20 blades, including 203 unmarred surfaces, 143 with scratches, and 48 with nicks. Examples are shown in Figure 2. Additionally, since no labels were originally provided, manual annotation was performed using CVAT, resulting in an updated dataset with 394 labeled images organized into corresponding images/ and label/ directories.

### 3.3. Bottle Defects Dataset Within a Control Environment

The following dataset is the first part of the dataset acquired as part of this paper. It introduces data related to pharmaceutical bottle defects acquired over a black background on a leveled surface. To streamline its reference throughout this study, this part of the dataset will be mentioned as the Bottle Defect Dataset in Controlled Environment (BDDCE). The dataset was designed to enable the development and evaluation of ML models for visual defect detection in pharmaceutical bottles, focusing on the defects and minimizing environmental and scenario effects. The pharmaceutical packaging produced by Neutroplast can be categorized into three distinct types. Type 1 corresponds to intermediate packaging with different lengths and widths, type 2 to standard packaging with a round body with a single radius, and type 3 to larger packaging with similar characteristics to type 2.

**Figure 3:**
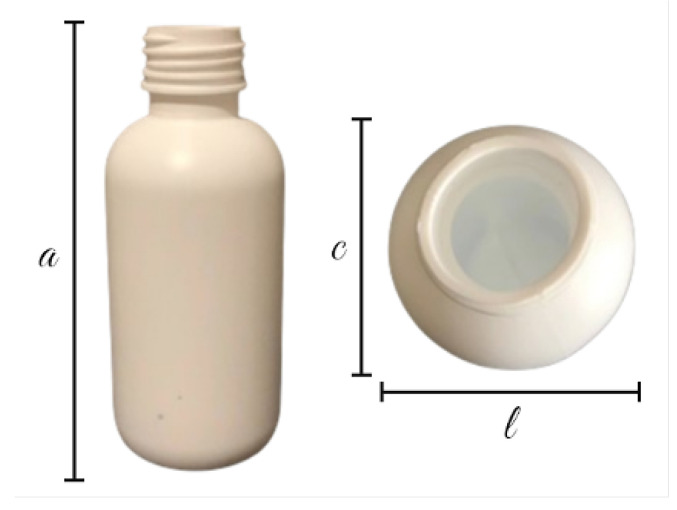
Typical dimensions of the types of pharmaceutical packaging produced by Neutroplast.

Regarding the dimensions of the selected packages, Figure 3 shows the parameters analyzed: *l* represents the width, *a* the height, and *c* the length of the package. These dimensions may change based on the type of packaging being produced and consequently influence both the acquisition system and the resulting image appearance. Regarding the dimensions of each of the packaging types, Table 1 presents the main values for each of the previously mentioned indicators.

**Figure 4:**
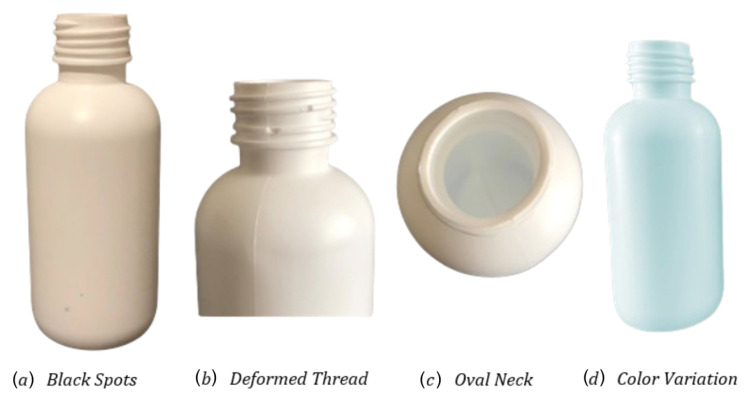
Example of defects on selected bottles.

Based on the industry operation and knowledge, a visual defect catalog was provided and defined in collaboration with Neutroplast to define common defects and their characterization. Subsequently, examples of products with defects were collected. Additional defects were manually introduced into other bottles to augment the dataset, with all modifications validated by operators. Figure 4 illustrates the implemented defects, which were classified into two groups: primary defects, representing the most frequent occurrences, and secondary defects.

A primary set of defects (considered of higher priority or more frequent) was identified. These include Deformed Thread, which is dependent on the degree of deformation and becomes more evident with pronounced cases, and Oval Neck, whose identification relies on the position of the neck of the bottle. Additional defects include Black Spots, which are considered only in white packaging due to the possibility of visual artifacts from alternative polymers concealing the defect, and Color Variation, which arises from differences in lighting conditions, especially in non-controlled environments. Regarding the secondary set of defects, their inclusion was conditional and of lower priority due to reduced frequency. These include Cut on Top of the Neck, which depends on the degree of the defect, and Flashes, included conditionally depending on severity, such as reflections caused by light.

**Figure 5:**
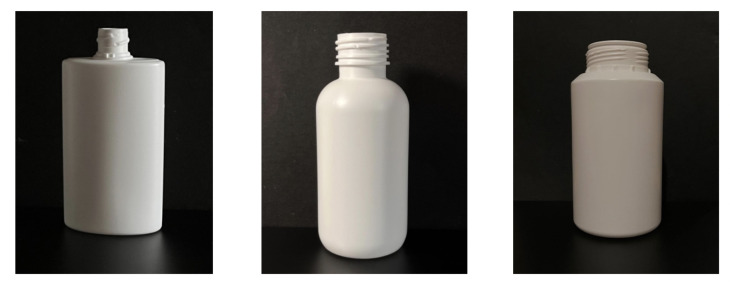
Examples from BDDCE: Type 1 (**left**), Type 2 (**center**), and Type 3 (**right**).

**Figure 6:**
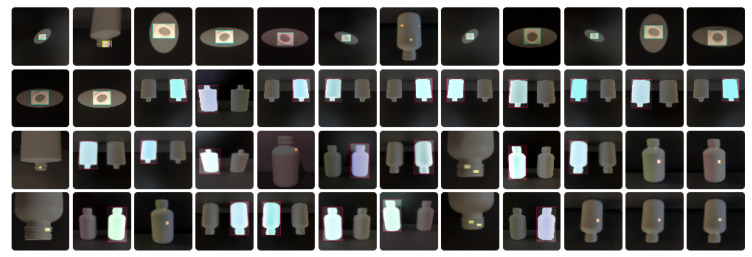
Example of annotations with bounding boxes on bottles with defects.

**Figure 7:**
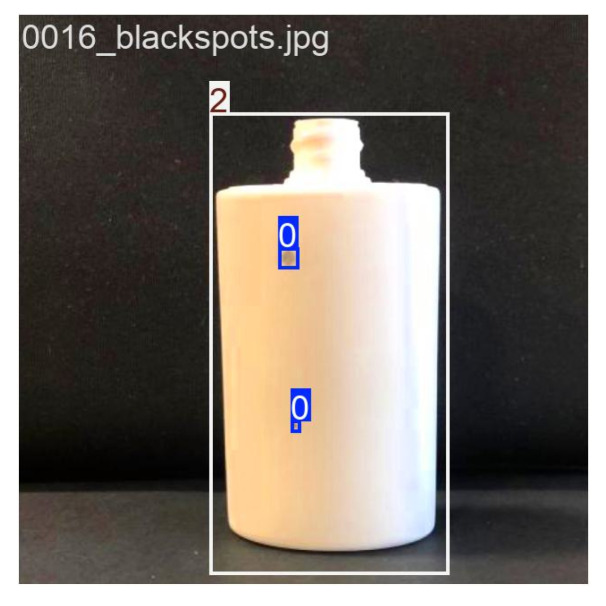
Visual representation of ground-truth annotations for bottle images, where class 0 represents the Blackspots defect and class 2 represents the Bottle category.

The image acquisition process was designed to ensure consistency and reliability in the dataset. Images were captured under controlled conditions, including adequate and varied lighting, the avoidance of direct light, text-free packaging, and close proximity to reduce background interference. The procedure involved setting up a contrasting black background, positioning the packaging in front of it, capturing images of both defective and non-defective objects, and organizing them into directories according to the defect type. The dataset images were captured using an iPhone 13, which features a 12 MP camera, a 26 mm focal length in standard mode, an f/1.6 aperture, and Optical Image Stabilization (OIS). Examples of the captured images are shown in Figure 5. Additionally, data augmentation techniques were applied to increase image variations, improve model robustness, and simulate diverse conditions, thereby enhancing generalization to new images. The transformations included a random vertical flip with a 100% probability, ensuring defects were recognized regardless of orientation, and random color jitter, which introduced variations in brightness, contrast, saturation, and hue (each set to ±20%) to mimic real-world variability. In addition, defects were manually annotated using the Computer Vision Annotation Tool (CVAT), in which bounding boxes were drawn around defects (as illustrated in Figure 6) and saved in text annotation files using the YOLO format structure (class_i, x_center, y_center, width, height). This process ensured precise labeling of defect locations, as illustrated in Figure 7.

**Figure 8:**
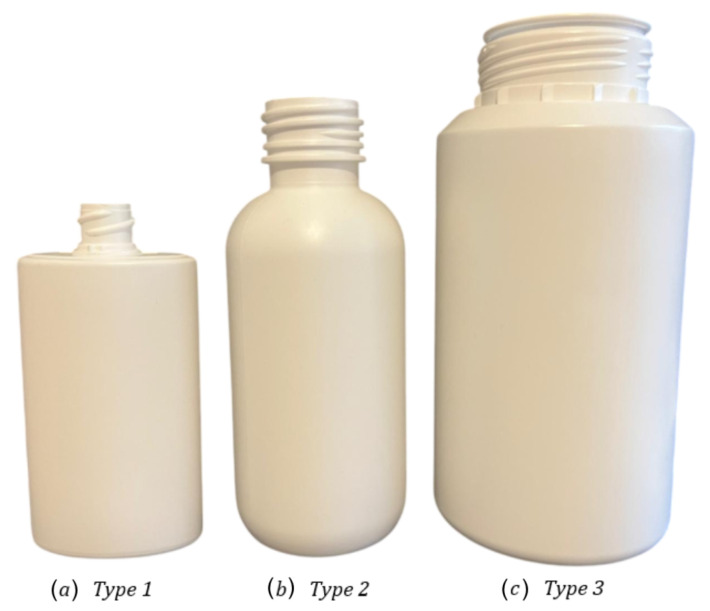
Types of plastic pharmaceutical bottles.

BDDCE consists of two main directories: images/, which contains all images of the plastic pharmaceutical bottles, and labels/, which contains the corresponding annotations for each image. In these directories, three folders (type1/, type2/, and type3/) are organized, representing three distinct types of plastic packaging, illustrated in Figure 8. This dataset comprises a total of 1852 images, with the class distribution presented in Table 2.

### 3.4. Bottle Defect Dataset Within Industrial Environment

This part of the dataset focuses on bottle defects acquired in a real industrial environment and will be mentioned from this point onwards as Bottle Defect Dataset in Industrial Environment (BDDIE), for ease of reference. BDDIE depicts real-world conditions that enable the development and evaluation of ML models, exposing them to industrial environmental challenges, which improves their ability to perform visual defect detection in pharmaceutical bottles. Moreover, the bottles used in BDDIE are of the same types and samples as those described in BDDCE, ensuring consistency between the datasets. However, BDDIE captures images under real industrial conditions, thereby providing a complementary set of data that reflects natural variability and environmental factors encountered during actual production.

**Figure 9:**
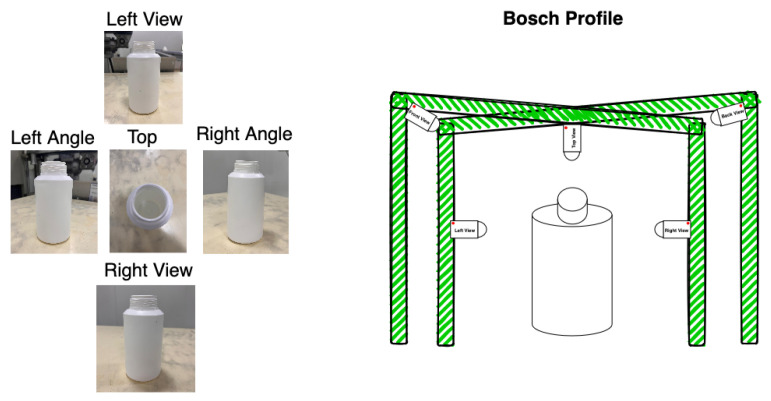
Available viewpoints (**left**) and proposed camera setup (**right**).

The images were captured under the following conditions: Five different viewpoints were used to simulate multi-camera setups—specifically, top, left, right, and two top-angled corners. These viewpoints were selected to closely replicate the configuration intended for deployment on the production line. The dataset includes a variety of lighting conditions and features text-free packaging. Each image was composed to include approximately 75% of the bottle in view, with the remaining portion capturing the surrounding industrial environment, thereby mimicking the camera positioning intended for the system to be deployed in production lines, illustrated in Figure 9.

Following the completion of the image capturing process in the industrial environment, data preparation was conducted, including image pre-processing, data augmentation techniques, and defect annotation. The final image dimensions were 1200 × 1600 pixels at 72 pixels per inch. Annotations of both the defects and the bottles were manually performed using the CVAT. Upon completion of the annotation process, data augmentation techniques were applied, including random color variations in the bottles, slight adjustments in brightness, and vertical flipping, as discussed previously in BDDCE.

The organizational structure of these datasets mirrors the structure described for BBDCE, consisting of two main directories, images/ and labels/, each containing three subfolders corresponding to the types of bottles, presented in Figure 8. This dataset contains a total of 2488 images, with the class distribution presented in Table 3.

## 4. Incremental Learning for Visual Inspection Proposal

The proposed solution focuses on integrating an intelligent, automated visual inspection module directly on the production line, structured around four main factors: multi-view cameras, image acquisition, a defect detection model, and decision support. Multi-view cameras are strategically positioned to capture images from multiple angles—top, front, back, and sides—ensuring full coverage of the packaging and detection of defects regardless of the location. Image acquisition can be performed in different ways, namely through a hardware trigger (e.g., an infrared cell), after which the captured image is sent to the defect detection model via a communication layer API for analysis. The defect detection model processes the images and, if a defect is identified, immediately notifies the actuator through the application layer, reducing the need for continuous human supervision. In cases of uncertainty, the operator is prompted via a graphical interface to confirm or reject the detection, allowing the system to iteratively refine the model over time. This decision support and user interaction create a self-improving system that progressively enhances detection accuracy while providing a transparent and responsive interface for real-time inspection and validation.

### 4.1. Data Distribution

A data distribution strategy was implemented during the model training process. Three different division schemes were applied to promote diversity and enhance the robustness of the evaluation, enabling a more versatile assessment of model performance. The following splits were applied to each dataset: 80% for training, 10% for validation, and 10% for testing; 70% for training, 20% for validation, and 10% for testing; and 70% for training, 10% for validation, and 20% for testing, as demonstrated and explained in [31,32].

### 4.2. Implementation Strategy

This subsection will discuss the implementation of both the baseline and incremental learning approaches, including the specific types of models and methodologies employed, as well as the rationale behind their selection.

#### 4.2.1. Baseline Approach

Establishing a baseline represents a fundamental step in developing the proposed automated visual inspection system, as it involves testing SOA models, defining the basis for objective comparison, and identifying the most suitable and robust model for the scenario. The evaluation covered five SOA models: Faster R-CNN (ResNet-50 backbone), YOLOv8 (nano and large versions), and YOLOv11 (nano and large), while one-stage and two-stage models were included to compare both types of approaches; examining YOLO’s nano and large variants provided further insights into the tradeoffs between model size, speed, and accuracy for deployment in industrial environments. All models were trained and evaluated on the four datasets using a 3-fold cross-validation approach to mitigate the risk of overfitting. Loss monitoring varied by model: YOLOv8 and v11 used Binary Cross-Entropy (BCE) for classification, Complete Intersection of Union (CIoU) for regression, and Distributed Focal Loss (DFL) for improved bounding box localization, whereas Faster R-CNN used cross-entropy for classification and smooth L1 loss for regression.

#### 4.2.2. Incremental Learning Approach

The incremental learning approach is intended for the model to learn and improve its knowledge over time by processing new data without retraining on the entire dataset. When further combined with LwF, it is capable of using the data from the new task to train its network while retaining past knowledge [7]. Such ability is deemed important in real-world industrial applications, where defect types may emerge over time and where frequent full retraining is impractical due to time constraints and computational costs.

This study aims to develop a model that consistently identifies the same defects while evaluating its performance whenever new defects are identified, reflecting real industrial conditions. This process involves applying LwF, which relies on the teacher-student framework. The teacher is the best-performing baseline, trained on a baseline subset of classes, referred to as old classes. The student model is then incrementally trained on the remaining classes, termed “new classes”, via LwF. LwF extends the Multiclass Cross-Entropy loss function by excluding indices related to old tasks, thereby incorporating a feedback mechanism from the prior model through knowledge distillation.

Evaluation was conducted using standard metrics such as precision, recall, and mAP, along with a modified KD loss (characterized in Equation (1)), to measure how well the student model was capable of retaining the teacher’s knowledge. The loss function is formulated as the aggregation of two distinct components: $L_{\mathrm{soft}}$ and $L_{\mathrm{hard}}$. The weighting coefficient α is applied only to the soft loss ($L_{\mathrm{soft}}$), therefore controlling the influence of the teacher, while the hard loss ($L_{\mathrm{hard}}$) ensures that the model is actually learning new features from ground truth data. Different α values (0.1,0.2,0.5,0.7,1) are used to explore varying contributions of teacher guidance, from minimal to equal to dominant influence.

(1) $$L_{\mathrm{KD}}\left(x\right)=\alpha \cdot L_{\mathrm{soft}}+L_{\mathrm{hard}}$$

With this approach, $L_{\mathrm{soft}}$ encourages the student to mimic the teacher’s predictions, whereas $L_{\mathrm{hard}}$ maintains standard supervised learning from actual labels.

The $L_{\mathrm{hard}}$ loss follows the standard YOLO approach, found in [33], calculating the student’s predictions for classification, bounding box regression, and objectiveness. It consists of three components: localization loss for bounding box inaccuracies, objectiveness loss for incorrect object presence predictions, and classification loss for errors in class probabilities. Conversely, $L_{\mathrm{soft}}$ contains two loss components: classification loss ($L_{\mathrm{LwF}\text{-}\mathrm{cls}}$) and regression loss ($L_{\mathrm{LwF}\text{-}\mathrm{reg}}$). These loss components were introduced into our LwF equation. For $L_{\mathrm{LwF}\text{-}\mathrm{cls}}$, Cross Entropy (CE) was deployed, as presented in Equation (2).

(2) $$L_{\mathrm{LwF}\text{-}\mathrm{cls}}\left(x\right)=-\sum_{i}^{k}{\hat{Y}}_{i}\cdot \log\left({\hat{X}}_{i}\right)$$

In Equation (2), ${\hat{Y}}_{i}$ represents the teacher’s logits over the k classes, and ${\hat{X}}_{i}$ is the logits obtained from the student model. Furthermore, the $L_{\mathrm{soft}}$ related to regression introduces a scaling factor for CE, referred to as the objectiveness score, as proposed in [26]. The bounding box logits from both the student and teacher models are normalized to improve numerical stability before computing the CE between them, as shown in Equation (3).

(3) $$L_{\mathrm{LwF}\text{-}\mathrm{reg}}\left(x\right)=-\sum_{i}\left({\hat{Y}}_{i}\cdot \log\left({\hat{X}}_{i}\right)\right)\cdot w_{k}^{\left(i\right)},\;\mathrm{where}\;w_{k}^{\left(i\right)}=\max\left(\mathrm{softmax}(t_{i})\right)$$

**Figure 10:**
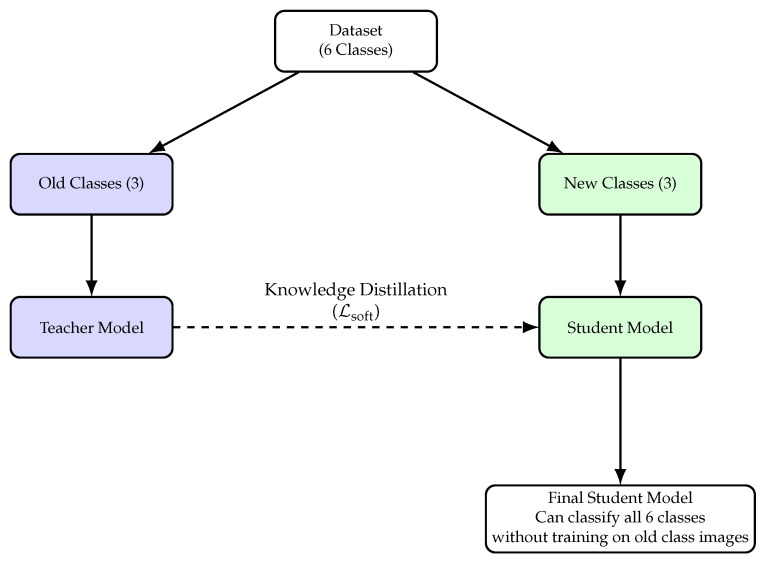
3 + 3 incremental learning workflow: old classes train the teacher, new classes train the student, and knowledge is transferred via distillation.

The final loss equation for incremental learning with LwF is illustrated in Equation (4). Additionally, LwF was evaluated in a 3 + 3 setting with the available data, which consists of dividing the dataset into old and new classes (3+3) as depicted in Figure 10. The old classes are used to train the teacher, whereas the new ones are used to train the student. This process mirrors real-world conditions, where initially little to no information is available, and additional data are gradually introduced over time. Moreover, at the end of this step, the student model is able to classify all 6 classes without ever ’seeing’ data from the old ones (teacher’s classes).

(4) $$L\left(x\right)=\alpha \cdot \left(L_{\mathrm{LwF}\text{-}\mathrm{reg}}\left(x\right)+L_{\mathrm{LwF}\text{-}\mathrm{cls}}\left(x\right)\right)+L_{\mathrm{YOLO}\text{-}\mathrm{hard}}\left(x\right)$$

As illustrated in Figure 10, the teacher model is first trained on the old classes. Subsequently, the student model is only trained on the new classes. During this later step, the teacher guides the student on the old classes through knowledge distillation, while the student simultaneously learns to detect the new classes. This step has the primary goal of showing the effectiveness of mitigating catastrophic forgetting. To implement this method, a modification to the $L_{\mathrm{YOLO}\text{-}\mathrm{hard}}\left(x\right)$ equation was made to enable the student model to retain past knowledge while learning new data. A soft mask was integrated to mitigate the influence of gradients from the old-class components in the classification component of $L_{\mathrm{YOLO}\text{-}\mathrm{hard}}\left(x\right)$. This approach drastically attenuates their contribution without completely nullifying it, facilitating the propagation of small gradients throughout the training process [34]. In contrast, a hard mask zeros out certain weights, blocking gradient flow in those areas, leading to catastrophic forgetting. Therefore, a soft mask, with β=0.1, was introduced by altering the classification component as shown in Equation (5).

(5) $$L_{\mathrm{YOLO}\text{-}\mathrm{cls}}\left(x\right)=\sum_{i=0}^{S^{2}}1_{i}^{\mathrm{obj}}\sum_{c\in \mathrm{classes}}\left[1_{c}^{\mathrm{new}}{\left(p_{i}\left(c\right)-{\hat{p}}_{i}\left(c\right)\right)}^{2}+\beta \cdot 1_{c}^{\mathrm{old}}{\left(p_{i}\left(c\right)-{\hat{p}}_{i}\left(c\right)\right)}^{2}\right]$$

In Equation (5), $1_{i}^{\mathrm{obj}}$ is an indicator function that equals 1 if an object is present in grid cell *i*, and 0 otherwise. $p_{i}\left(c\right)$ and ${\hat{p}}_{i}\left(c\right)$ represent the predicted and annotated class logits for class *c* at *i*, respectively. $1_{i}^{\mathrm{new}}$ and $1_{i}^{\mathrm{old}}$ represent binary masks used to distinguish between new and previously learned (old) classes.

### 4.3. Performance Metrics

The metrics selected for monitoring the training and evaluation processes were precision, recall, mAP@50, and mAP@50-95, targeting the performance when detecting objects. The precision metric (Equation (6)) enables us to determine, from all the objects the model predicted, how many were actually correct [35].

(6) $$Precision=\frac{TP}{TP+FP}$$

Following this equation, TP (True Positives) represents the number of correctly detected defective instances, and FP (False Positives) identifies the number of non-defective instances that were incorrectly identified.

As for the recall metric (Equation (7)), it measures how many actual objects in the image the model correctly detected. This indicates that when recall is high, the model is able to detect most of the relevant objects, minimizing missed detections [36]. In this study, recall is considered the primary metric of interest, as it plays a critical role in detecting anomalies on the production line. It is important to detect all potential defects, even at the risk of some FP, rather than to miss defects entirely. The goal is to ensure that any possible issue is flagged for review, rather than allowing a defective product to go unnoticed, which is crucial within the industrial setting and ensures more adequate usage with decision support. Therefore, a higher weight is given to recall when evaluating the performance of the model.

(7) $$Recall=\frac{TP}{TP+FN}$$

Furthermore, mAP (Equation (8)) is utilized to evaluate the performance of object detection models by averaging the precision-recall curves for each object class [35].

(8) $$mAP=\frac{1}{N}\sum_{k=1}^{k=n}AP_{k}$$

In this case, where *N* is the total number of classes, and $AP_{k}$ is the Average Precision computed for the *k* class. $AP_{k}$ can be further analyzed through the subsequent equation [37]:

(9) $$AP_{k}=\sum_{k=0}^{n-1}\left(\mathrm{recalls}(k)-\mathrm{recalls}(k+1)\right)\cdot \mathrm{precisions}\left(k\right)$$

In this equation, *n* is the total number of thresholds, with recalls(n)=0 and precisions(n)=1.

Inference and training times were evaluated to assess the performance efficiency of each model. Inference time measures how quickly a model can process images, which is crucial for real-time inspection in industrial settings. The training time also plays an important role, as it reflects the efficiency of the training process and indicates how quickly a model can be trained or updated with new data. In the target scenarios, a small training time translates into reduced stops of the production line and consequently higher product throughput. Usage of these metrics allows comparison between models in terms of speed and resource requirements [38].

The evaluations were conducted on different hardware configurations. For baseline experiments, the Tesla V100-SXM2-32 GB (manufacturer: NVIDIA, Santa Clara, CA, USA), NVIDIA A100 80 GB PCIe (manufacturer: NVIDIA), and Quadro RTX 6000 24 GB GPUs (manufacturer: NVIDIA) were used. Incremental learning experiments were performed on an NVIDIA GeForce GTX 1080 GPU (manufacturer: NVIDIA). Reporting hardware alongside performance metrics provides context for industrial deployment, helping to assess whether a model can meet real-time constraints and the computational resources required for training and inference.

## 5. Results and Discussion

This section presents and analyzes the results from baseline and incremental learning experiments, identifying the best models, evaluating data division strategies, comparing outcomes with initial objectives, and discussing challenges, resolutions, and critical insights.

### 5.1. Baseline Models

To establish performance benchmarks, baseline experiments were carried out with five object detection models trained on the prepared datasets using varying epochs and batch sizes, enabling a detailed comparison of how these factors impact metrics like mAP, recall, and precision.

#### 5.1.1. YOLOv8

Each of the YOLOv8 models was subjected to evaluation using the aforementioned performance metrics, with the best set of hyperparameters selected based on the highest recall on the test set. Overall results are summarized in Table 4.

On BDDCE, YOLOv8 large consistently outperformed YOLOv8 nano across all dataset distributions, with the best performance being achieved using larger batch sizes and the 70_20_10 split, using the Tesla V100-SXM2-32 GB GPU (manufacturer: NVIDIA). Nevertheless, YOLOv8’s large version achieved a slightly higher recall (94% vs. 93.6%), while YOLOv8 nano retained clear advantages in training and inference speed. With regard to BDDIE, both YOLOv8 nano and YOLOv8 large achieved strong results under realistic conditions, with YOLOv8 large reaching 93.6% recall and YOLOv8 nano achieving 92.8% in the 80_10_10 split, using Quadro RTX 6000 24 GB GPU (manufacturer: NVIDIA). YOLOv8 nano demonstrated better computational efficiency, delivering considerably faster training (0.183 vs. 0.5 h) and inference times (3.1 ms vs. 23.6 ms). Furthermore, on the MSDD, YOLOv8 nano outperformed YOLOv8 large across all distributions, peaking at 95.7% recall compared to YOLOv8 large’s 90.2%, using NVIDIA A100 80 GB PCIe GPU (manufacturer: NVIDIA). This reflects the YOLOv8 nano model’s better generalization on unbalanced data, supported by DFL, alongside its faster training and inference speeds. Conversely, on the BSDD, recall was lower overall, with nano achieving 76.6% compared to large’s 71.5%, using a Tesla V100-SXM2-32 GB GPU (manufacturer: NVIDIA). YOLOv8 nano generalized better under scarce data, while YOLOv8 large struggled with higher training losses, though YOLO’s built-in DFL helped stabilize outputs, and YOLOv8 nano again showed faster training and inference speeds.

#### 5.1.2. YOLOv11

The YOLOv11 models were also evaluated using the previously defined metrics. The best-performing selection of hyperparameters was determined based on the highest recall obtained with the test set. As shown in Table 5.

On BDDCE, YOLOv11 large consistently outperformed YOLOv11 nano in recall, peaking at 92.9% (70_20_10) vs. nano’s 92.8%, using Quadro RTX 6000 24 GB GPU (manufacturer: NVIDIA), though YOLOv11 nano was faster in training (0.13 h vs. 0.37 h) and inference (2.6 ms vs. 20.3 ms). As for BDDIE, both models generalized well, with YOLOv11 large reaching 92.9% recall (70_10_20), using NVIDIA A100 80 GB PCIe GPU (manufacturer: NVIDIA), and YOLOv11 nano 92% (80_10_10) using Quadro RTX 6000 24 GB GPU (manufacturer: NVIDIA), while YOLOv11 nano remained faster in training (0.162 h vs. 0.582 h) and inference (3 ms vs. 17 ms). Moreover, on the MSDD, YOLOv11 nano slightly outperformed YOLOv11 large, achieving 95.2% recall (70_10_20) vs. 93.4% (80_10_10) using Tesla V100-SXM2-32 GB GPU (manufacturer: NVIDIA), while also being quicker in training (0.09 h vs. 0.21 h) and inference (3 ms vs. 21.1 ms). Furthermore, on the BSDD, both models showed lower recall due to limited data, with YOLOv11 nano leading at 77.3% (70_20_10) compared to YOLOv11 large at 73.2%, using Tesla V100-SXM2-32 GB GPU (manufacturer: NVIDIA), and also maintaining shorter training times (0.021 h vs. 0.095 h) and faster inference (3.6 ms vs. 21 ms).

#### 5.1.3. Faster R-CNN

The Faster R-CNN model was also evaluated with the metrics described earlier, with the best-performing selection of hyperparameters being determined based on the highest recall observed on the test set. Table 6 presents these results.

On BDDCE, the faster R-CNN model delivered stable performance with 50 epochs, achieving the highest recall of 77.1% on the 70_20_10 distribution, using a Tesla V100-SXM2-32 GB GPU (manufacturer: NVIDIA), though at the cost of longer training times (7+ h) and inference times, which averaged 69 ms. With regard to BDDIE, this model stabilized at 50 epochs with the best recall of 72.1% (70_20_10), using a Tesla V100-SXM2-32 GB GPU (manufacturer: NVIDIA), requiring 6.5 h of training and 69 ms for inference, which would have a negative impact in terms of production line stops for retraining. The unbalanced MSDD model achieved its best recall of 92.9% on the 70_10_20, using Tesla V100-SXM2-32 GB GPU (manufacturer: NVIDIA), distribution at 50 epochs, though class imbalance led to low precision; training averaged 3 h with 68 ms inference. Lastly, with BSDD, the best recall was 86.9% (70-10-20) at 50 epochs, using a Tesla V100-SXM2-32 GB GPU (manufacturer: NVIDIA), with 1.2 h of training and inference time of around 68 ms.

### 5.2. Incremental Learning

The following section presents the application of incremental learning to the YOLOv8 nano model, chosen for its faster training and inference compared to YOLOv8 large and for being considered a stable proposition when compared to YOLOv11. An ablation study is first conducted to determine the optimal α value for knowledge distillation, followed by validation to confirm proper incremental learning behavior. Once validated, an additional ablation study was conducted to select the best learning rate, which was then applied to a series of 1–10 incremental learning simulations in order to evaluate the model’s performance across sequential stages.

#### 5.2.1. Knowledge Distillation

An ablation study was conducted to evaluate the impact of Knowledge Distillation (KD) on incremental learning by varying the distillation weight, α, using YOLOv8 nano as both teacher and student. The teacher was selected from the baseline BDDCE experiments (50 epochs, with a batch size of 8 images) due to its computational efficiency and strong validation performance. All simulations were conducted using the same training settings in order to provide a reference baseline for subsequent tests. Table 7 presents the results of the teacher model. The model performed well on certain defects, such as color variation, but struggled with black spots. This limitation can be attributed to an insufficient number of samples per class, but in this particular case, insufficient resolution plays a critical role. Given the position of the bottles and the image resolution, this particular type of defect translates into very small regions, often of just a couple of pixels.

**Figure 11:**
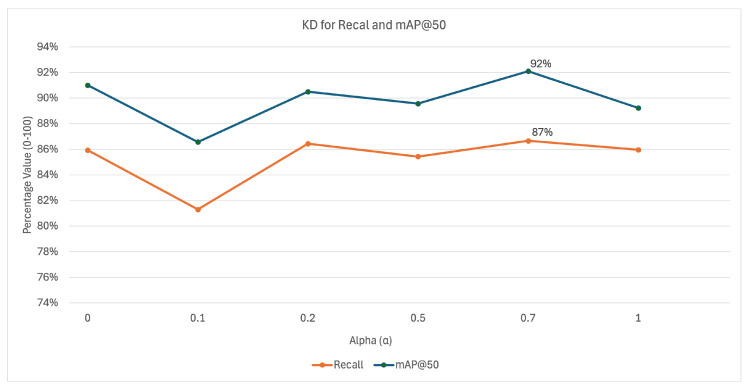
KD results for recall and mAP@50 for varying α values (0–1).

Furthermore, KD training was performed on BDDCE at various α values (0, 0.1, 0.2, 0.5, 0.7, and 1) with 3-fold cross-validation to determine the optimal setting for incremental learning, allowing us to analyze and determine the degree of impact on the student model from the teacher’s influence. Figure 11 shows the average recall and mAP@50 for each α.

Additionally, the KD studies have shown that model performance varies according to the α value: at α=0, no distillation occurs and losses are higher, while small values like α=0.1 reduce classification and regression losses by leveraging soft targets. As α increases from 0.2 to 0.5, teacher influence strengthens and performance stabilizes, but very high values (α=0.7–1) risk underfitting by limiting the student’s ability to learn new features. Overall, α=0.7 provided the best balance, achieving the highest recall, and was selected for all subsequent simulations.

#### 5.2.2. Incremental Learning Validation

After selecting the optimal α value, incremental learning was validated using a modified BDDCE dataset with the first three classes (Black Spots, Color Variation, and Bottle). These classes were chosen to represent both defects (e.g., black spots, color variation) and structural features (bottle shape) that are critical in real production environments. The teacher model, a YOLOv8 nano, was trained for 50 epochs with a batch size of 8 using 3-fold cross-validation. The results obtained are summarized in Table 8.

The teacher model selected for incremental learning was obtained in fold 2, achieving the highest recall of 91%. Using this teacher, the YOLOv8 nano student model was trained on all six classes with α=0.7 for 50 epochs and a batch size of 8 images on the 70_20_10 distribution, while monitoring student, teacher, classification, and regression losses. Evaluation results are shown in Table 9.

As demonstrated in Table 9, the incremental learning approach (LwF) is deemed effective, as the color variation class achieves 100% recall and 99% mAP@50, and the bottle class reaches 99.6% recall and 99.2% mAP@50, demonstrating successful knowledge retention. Moreover, the model successfully learned the new classes, achieving, for example, oval neck with a recall of 91.9% and mAP@50 of 93.7%. However, the black spots class showed a 46.7% recall compared to the teacher’s 73.9%. This drop can be attributed to several factors: (i) the limited number of training samples for this class, which restricts generalization; (ii) the small size of black spots, which makes them inherently difficult to detect; and (iii) the fact that these defects are sensitive to variations in lighting and resolution, meaning that lower-quality images make it harder to detect. To mitigate these challenges, potential solutions include acquiring higher-quality images to reduce noise and improve the visibility of small defects. In addition, applying prediction-wise weighting can emphasize harder-to-detect defects, while multi-scale architectures can enhance feature extraction across resolutions, improving the detection of fine-grained patterns. These approaches represent promising directions for future work.

#### 5.2.3. Learning Rate Study

After validating incremental learning with LwF, an ablation study was performed to select an appropriate learning rate for the simulations, as the previous rate used for the teacher and baseline models would be inefficient for the smaller batches of 50 images per class. Table 10 presents the results for the teacher model trained for 50 epochs with a batch size of 8 images on an NVIDIA GeForce GTX 1080 GPU (manufacturer: NVIDIA).

Four different learning rates ($3\times 10^{-4},1\times 10^{-4},5\times 10^{-5},1\times 10^{-5}$) were tested to determine the optimal rate for the simulations using the following procedure: The teacher model was initially trained with 50 images per class, and, subsequently, the best-performing model from the previous cycle was used as the student for the next cycle, using a neutral α of 0.5. The most stable recall values and best results across 21 cycles were monitored, where each cycle introduces a new batch of 50 random images for training the student model. Table 11 presents the optimal learning rate.

The learning rate of $5\times 10^{-5}$ was determined to be the optimal setting, allowing us to achieve the highest recall of 81.3% after 21 cycles while also showing consistent improvement in student, classification, and regression losses throughout the simulation.

#### 5.2.4. Simulations

A series of 21 simulation cycles was conducted to evaluate the previously identified incremental learning setup across 10 task sequences, focusing on challenges such as class imbalance, limited data, domain shifts, and emerging defects. The experiments were conducted using an NVIDIA GeForce GTX 1080 GPU (manufacturer: NVIDIA), which relied on images extracted from the BDDCE and BDDIE datasets. The model was trained for 50 epochs, with a batch size of 8 images, α=0.7, and a learning rate of $5\times 10^{-5}$. The teacher model, based on YOLOv8 nano, was trained under three configurations: 50 images per class for all classes, 50 images per class for the first three classes only (old classes), and on the full dataset.

**Figure 12:**
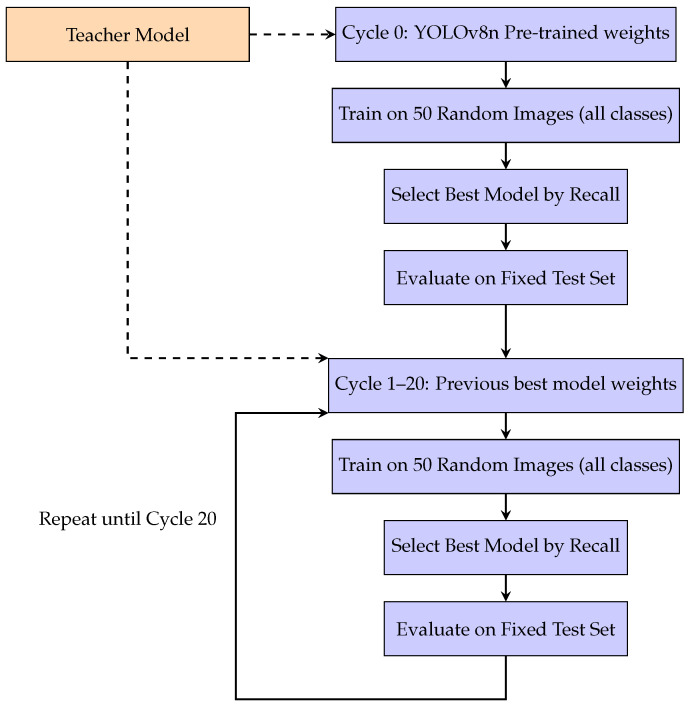
Incremental learning process showing the teacher model providing guidance for cycle 0 and cycles 1–20. Dashed arrows indicate knowledge distillation from the teacher.

During training, the student model starts with the predefined YOLOv8 nano weights and is initially trained on 50 randomly selected images per class, consistent across datasets (BDDCE or BDDIE). The test set remains fixed, using the same images employed for evaluating the teacher model to ensure reliable comparisons. Over 21 incremental learning cycles, each cycle adds a new batch of 50 images for student training. The student model’s best weights, guided by the teacher via KD, are saved at the end of each cycle and carried over to the next. After evaluation on the fixed test set, the training images from the cycle are removed to prevent overlap with future training. This process is demonstrated in Figure 12.

The following list explains each experiment, serving as a brief table of contents for the upcoming experiments:

- Experiment 1: BDDCE—All Classes
  The first experiment simulates low-resource industrial conditions by introducing only 50 images per class on BDDCE.
- Experiment 2: BDDCE—First, 3 Classes
  This experiment also simulates low-resource conditions using BDDCE but limits training to only 50 images from the first three classes, with the teacher model trained solely on these classes.
- Experiment 3: BDDIE—All Classes
  This experiment mirrors the setup of experiment 1 but uses BDDIE for both training and testing, with 50 images per class. The teacher model is trained on BDDIE using all classes.
- Experiment 4: BDDIE—First, 3 Classes
  Similarly to experiment 2, this experiment uses BDDIE instead of BDDCE, training on only 50 images from the first three classes. The teacher model is trained on BDDCE using only the first three classes.
- Experiment 5: BDDCE + BDDIE—All Classes
  This experiment introduces images from both BDDCE and BDDIE for training (50 images per class each), while testing is conducted exclusively on BDDIE. The teacher model is trained using both datasets.
- Experiment 6: BDDCE + BDDIE—First, 3 Classes
  This experiment builds on experiment 5 by limiting training to only the first three classes from both BDDCE and BDDIE (50 images each), with testing still conducted on BDDIE.
- Experiment 7: BDDCE—Baseline—α=0.7
  This experiment evaluates the best-performing baseline teacher model trained on the BDDCE on BDDIE.
- Experiment 8: BDDCE—First, 3 Classes Baseline—α=0.7
  Using the teacher model from the incremental learning validation experiment, this experiment simulates a scenario where only three known classes were trained in a controlled setting on BDDCE and evaluated on BDDIE.
- Experiment 9: BDDCE—Baseline—α=0.2
  This experiment follows the setup of experiment 7 but introduces a distillation weight of α=0.2 to reduce the teacher’s influence due to its poor performance.
- Experiment 10: BDDCE—First, 3 Classes Baseline—α=0.2
  Similarly to experiment 8, this experiment applies a lower distillation weight of α=0.2 to mitigate the impact of a weak teacher model trained on only three classes.

In this study, the results obtained when training the proposed incremental learning method with LwF were consistent with expectations. As shown in Table 12, both recall and mAP@50 improved notably from the first to the final cycle across all experiments. Appendix A provides graphical representations illustrating how recall and mAP@50 evolved over the cycles in each experiment.

### 5.3. Discussion

The results from both the baseline evaluations and the incremental learning simulations demonstrate the effectiveness of developing an automated visual defect detection system for industrial applications, highlighting the strengths and weaknesses of various AI-based ML models for object detection while also demonstrating the advantages of incremental learning in dynamic scenarios.

In the baseline experiments, statistical significance testing and confidence intervals were not performed. Although cross-validation was conducted to assess model performance across different distributions, the reported results represent single observed values for each metric. Moreover, YOLOv8 and YOLOv11 generally showed strong performance across datasets, especially in recall and mAP@50. YOLOv8 and YOLOv11 large performed better on well-balanced datasets such as BDDCE and BDDIE by learning more complex features, but at the cost of longer training and inference times. In contrast, YOLOv8 and YOLOv11 nano models offered faster inference, making them preferable for real-time use. Interestingly, with imbalanced datasets (MSDD) or limited samples per class (BSDD), YOLOv8 and YOLOv11 nano models outperformed the YOLOv8 and YOLOv11 larger models, likely due to their more efficient architectures, as both variants integrate features like DFL, which help mitigate class imbalance; while the YOLO models achieved high precision (around 90%), our baseline analysis focused on recall as the decisive metric. In industrial inspection, it is more critical to flag all potential defects, ensuring that every possible issue undergoes review, rather than risk allowing a defective product to go unnoticed. Moreover, Faster R-CNN performed well on structured datasets but was hindered by longer processing times and difficulties with unbalanced data, showing high recall but low precision due to the lack of focal loss. To address these limitations, the implementation of focal loss is recommended, as it emphasizes harder-to-learn classes rather than disproportionately focusing on the easier ones.

The incremental learning simulations with LwF have demonstrated a progressive increase in performance results, verifying that the combination of incremental learning with LwF is successful, enabling the student model to retain prior knowledge while also learning new knowledge. The ablation experiment with KD’s α parameter was significant in that a value of 0.7 yielded the most adequate results, with the highest recall, and it was subsequently utilized for the incremental learning simulations. Across diverse conditions—including sparse data, real-world industrial environments, and mixed datasets—the student model consistently reduced loss while improving recall and mAP@50 over 21 incremental learning cycles. This demonstrates that the model can effectively learn from small batches of data without disrupting the production process. Compared to retraining a teacher model from scratch, incremental learning required significantly less training time while maintaining similar inference speeds, highlighting its practicality for industrial applications where full retraining is often impractical.

Additionally, when the teacher was trained solely on clean, uniform datasets like BDDCE, it struggled to generalize to real-world environment data (BDDIE), a common issue with overfitting. In such cases, the student model outperformed the weak teacher in all critical metrics. Experiments showed that a lower α value (α=0.2) allowed the student greater autonomy to learn directly from real data while still leveraging the teacher’s prior knowledge. Using this setting, in Experiment 10, the student achieved a recall of 72.9% and mAP@50 of 73.8%, which is slightly lower than the teacher’s 83.6% recall and 80.9% mAP@50. Despite this, the student continued to learn from small batches over time, retaining knowledge from previous cycles, thereby demonstrating the practical benefits of incremental learning for industrial inspection. This approach was particularly beneficial when the teacher was trained in a different domain. However, detecting smaller defects, such as black spots and deformed threads, remained challenging, as the possibility of fewer samples was introduced at each cycle. Smaller defects required more samples to generalize compared to larger defects and their low-resolution appearance in the images. For example, a black spot originally measured approximately 15 × 15 pixels; after resizing, it was reduced to roughly 7 × 7 pixels, making it even more difficult for the model to distinguish. To address this, higher-resolution images should be introduced during training. Methods such as prediction-wise weighting [26] could help the model focus on smaller defects, such as black spots, which are harder to detect. Meanwhile, cross-modal representation learning [39,40] could improve knowledge transfer between the student and teacher. Such approaches hold potential for surpassing both the teacher models used here and the large models from the baseline experiments.

## 6. Conclusions

This research work addressed the shortcomings of manual inspection in industrial environments, where the process is considered laborious, unreliable, and limited by human fatigue, while also addressing the challenge of scarce data availability. To overcome these issues, an automated visual inspection system was developed using AI and CV, centered on the use of incremental learning with LwF to enable continuous improvement without requiring retraining. Additionally, datasets of pharmaceutical bottles were developed, along with the use of external datasets, highlighting the importance of large, high-quality data for effective training. Models such as YOLOv8, YOLOv11, and Faster R-CNN were trained and evaluated, with incremental learning proving to be effective in mitigating catastrophic forgetting and allowing the student model to retain knowledge while acquiring new defect classes. Results from baseline experiments and incremental learning simulations showed steady improvements in recall, mAP@50, and reduced training loss, while maintaining shorter training times and reliable inference rates.

Visual inspection within an industrial production line presents a set of specific challenges and requirements, including the need for fast processing aligned with the product passing rate, physical constraints, limited hardware for processing, the need to minimize the rate of unidentified defects, and the lack of a full, representative dataset at the start. The proposed work addresses these challenges through the use of models with small inference and high recall, but above all, through their combination with an incremental learning strategy. Despite challenges in detecting smaller defects, the system demonstrated adaptability and scalability. By collecting user feedback, the proposed solution demonstrated adaptability by incrementally incorporating new examples of defects, as well as future unseen defects, thereby alleviating the burden of an initial large dataset. This incremental learning strategy also implies shorter training times, which can result in shorter stops of the production line for updates.

## Figures and Tables

**Table 1:** Dimensions of the different types of packaging selected.

Packaging Type	Width (cm)	Height (cm)	Length (cm)
Type 1	4.45	9	2.45
Type 2	4.5	11.5	4.5
Type 3	6.5	13.3	6.5

**Table 2:** Defect categories in the controlled environment dataset.

Defect Type	Number of Images
Black spots	374
Color variation	367
Deformed thread	368
Oval neck	372
No defect	371

**Table 3:** Defect categories in the industrial environment dataset.

Defect Type	Number of Images
Black Spots	499
Color Variation	512
Deformed Thread	499
Oval Neck	494
No Defect	484

**Table 4:** Performance from YOLOv8 models for each dataset across all data splits, including top-performing settings per distribution.

Dataset	Model	Split	Epochs	Batch	Training Time (h)	Class Loss	Box Loss	Precision	Recall	mAP@50	mAP@50-95	Inference (ms)
BDDCE	YOLOv8n	80/10/10	50	32	0.338	0.367	0.237	0.917	0.896	0.912	0.731	2.8
		70/20/10		64	0.106	0.368	0.243	0.917	**0.936**	0.938	0.752	2.8
		70/10/20		32	0.063	0.370	0.248	0.924	0.896	0.905	0.729	2.8
	YOLOv8l	80/10/10	50	32	0.338	0.373	0.212	0.915	0.914	0.913	0.733	23.6
		70/20/10		16	0.353	0.371	0.209	0.919	**0.940**	0.945	0.759	23.1
		70/10/20		8	0.193	0.380	0.233	0.914	0.916	0.921	0.738	22.6
BDDIE	YOLOv8n	80/10/10	50	16	0.147	0.60293	0.35877	0.912	**0.928**	0.927	0.726	2.8
		70/20/10		8	0.214	0.60480	0.38183	0.889	0.914	0.928	0.737	2.8
		70/10/20		8	0.189	0.60915	0.38136	0.899	0.915	0.922	0.722	2.8
	YOLOv8l	80/10/10	50	8	0.532	0.58354	0.33198	0.932	**0.936**	0.953	0.744	23.7
		70/20/10		16	0.490	0.58677	0.32988	0.924	0.929	0.958	0.750	23.3
		70/10/20		8	0.469	0.73761	0.60451	0.813	0.840	0.858	0.645	11.0
MSDD	YOLOv8n	80/10/10	50	8	0.084	0.23885	0.27360	0.907	**0.957**	0.948	0.739	2.8
		70/20/10		32	0.078	0.28267	0.28311	0.879	0.927	0.928	0.653	2.8
		70/10/20		32	0.067	0.21248	0.25980	0.870	0.940	0.924	0.695	2.7
	YOLOv8l	80/10/10	50	16	0.253	0.22931	0.24993	0.605	0.482	0.490	0.340	24.2
		70/20/10		16	0.174	0.37128	0.22089	0.901	0.895	0.897	0.584	24.2
		70/10/20		32	0.223	0.22072	0.25048	0.833	**0.902**	0.893	0.649	11.8
BSDD	YOLOv8n	80/10/10	50	64	0.074	0.78502	0.85533	0.737	0.679	0.621	0.470	3.1
		70/20/10		8	0.051	0.74818	0.88053	0.500	**0.766**	0.563	0.421	3.0
		70/10/20		32	0.062	0.84231	0.90273	0.589	0.642	0.568	0.433	3.1
	YOLOv8l	80/10/10	50	8	0.111	0.91288	1.02700	0.474	0.703	0.602	0.453	23.5
		70/20/10		8	0.113	0.90840	0.97775	0.461	**0.715**	0.573	0.397	23.7
		70/10/20		32	0.120	0.80387	0.72388	0.586	0.652	0.579	0.435	23.8

Note: Bold values in the recall column indicate the highest performance achieved for each model per dataset.

**Table 5:** Best performing results from YOLOv11 for each dataset across all data splits, showing the optimal settings per distribution.

Dataset	Model	Split	Epochs	Batch	Training Time (h)	Class Loss	Box Loss	Precision	Recall	mAP@50	mAP@50-95	Inference (ms)
BDDCE	YOLOv11n	80/10/10	50	16	0.162	0.37530	0.23405	0.889	0.909	0.894	0.728	2.7
		70/20/10		32	0.119	0.36844	0.23969	0.928	**0.928**	0.929	0.744	2.5
		70/10/20		64	0.098	0.36493	0.25005	0.916	0.902	0.913	0.727	2.1
	YOLOv11l	80/10/10	50	32	0.382	0.38924	0.22975	0.913	0.903	0.907	0.727	20.3
		70/20/10		16	0.353	0.39942	0.23687	0.918	**0.929**	0.941	0.747	20.3
		70/10/20		8	0.365	0.39520	0.23602	0.877	0.908	0.910	0.732	20.3
BDDIE	YOLOv11n	80/10/10	50	32	0.158	0.60507	0.35816	0.913	**0.920**	0.936	0.739	2.7
		70/20/10		8	0.251	0.60995	0.38729	0.909	0.914	0.925	0.724	2.7
		70/10/20		32	0.076	0.60327	0.36607	0.904	0.906	0.920	0.725	3.1
	YOLOv11l	80/10/10	50	8	0.508	0.60285	0.35625	0.889	0.928	0.939	0.727	15.2
		70/20/10		32	0.450	0.60907	0.36075	0.905	0.920	0.944	0.745	15.3
		70/10/20		16	0.402	0.63718	0.45083	0.886	**0.929**	0.926	0.724	21.2
MSDD	YOLOv11n	80/10/10	50	16	0.110	0.20390	0.27131	0.858	0.945	0.951	0.709	3.1
		70/20/10		32	0.082	0.20086	0.24453	0.790	0.901	0.892	0.621	3.0
		70/10/20		32	0.078	0.21455	0.26217	0.878	**0.952**	0.951	0.698	3.0
	YOLOv11l	80/10/10	50	16	0.250	0.21000	0.23468	0.881	**0.934**	0.945	0.677	21.1
		70/20/10		32	0.241	0.28630	0.27654	0.866	0.890	0.911	0.617	21.0
		70/10/20		16	0.133	0.21832	0.28172	0.838	0.927	0.923	0.687	21.1
BSDD	YOLOv11n	80/10/10	50	64	0.031	0.78892	1.07391	0.606	0.654	0.611	0.457	3.7
		70/20/10		8	0.059	0.74901	0.92073	0.509	**0.773**	0.564	0.417	3.8
		70/10/20		16	0.021	0.85119	0.99043	0.605	0.626	0.541	0.422	3.5
	YOLOv11l	80/10/10	50	32	0.086	1.05403	0.90116	0.522	0.704	0.599	0.469	20.8
		70/20/10		32	0.098	0.80929	0.75795	0.502	**0.732**	0.554	0.414	20.9
		70/10/20		8	0.102	0.93782	0.95878	0.514	0.627	0.508	0.408	21.0

Note: Bold values in the recall column indicate the highest performance achieved for each model per dataset.

**Table 6:** Faster R-CNN results for each dataset across all data splits, including the top-performing selection of hyperparameters per distribution.

Dataset	Model	Split	Epochs	Batch	Training Time (h)	Class Loss	Box Loss	Precision	Recall	mAP@50	mAP@50-95	Inference (ms)
BDDCE	Faster R-CNN	80/10/10	10	16	1.03	0.058	0.053	0.3891	0.8120	0.5993	0.4967	50.11
			50	16	4.53	0.032	0.027	0.6320	0.7540	0.6910	0.6100	50.35
	Faster R-CNN	70/20/10	10	32	1.58	0.070	0.066	0.3038	0.8679	0.5847	0.4449	69.39
			50	16	7.133	0.034	0.028	0.5855	**0.7710**	0.6765	0.5879	69.93
	Faster R-CNN	70/10/20	10	16	1.067	0.057	0.053	0.3680	0.7946	0.5801	0.4790	69.84
			50	16	7.0167	0.032	0.028	0.6081	0.7449	0.6748	0.5884	68.31
BDDIE	Faster R-CNN	80/10/10	10	16	1.37	0.0587	0.0544	0.2528	0.7487	0.5001	0.4019	68.92
			50	8	6.72	0.0157	0.0194	0.7054	0.7095	0.7055	0.6075	68.61
	Faster R-CNN	70/20/10	10	16	1.53	0.0550	0.0550	0.2378	0.7557	0.4960	0.3947	68.86
			50	8	6.93	0.0160	0.0200	0.6511	**0.7210**	0.6842	0.5838	68.65
	Faster R-CNN	70/10/20	10	16	1.18	0.0560	0.0580	0.2303	0.7655	0.4972	0.3938	50.90
			50	8	5.83	0.0160	0.0200	0.6480	0.7170	0.6800	0.5700	51.05
MSDD	Faster R-CNN	80/10/10	10	8	0.63	0.020	0.025	0.4485	0.9370	0.6905	0.6495	68.36
			50	8	2.83	0.008	0.007	0.6290	0.9260	0.7740	0.7280	68.60
	Faster R-CNN	70/20/10	10	8	1.01	0.022	0.022	0.2558	0.9245	0.5889	0.5 401	68.27
			50	16	5.42	0.012	0.010	0.3425	0.9209	0.6300	0.5834	68.50
	Faster R-CNN	70/10/20	10	8	0.82	0.026	0.027	0.2570	0.9270	0.5910	0.5480	67.97
			50	16	2.58	0.013	0.010	0.5050	**0.9290**	0.7150	0.6730	68.46
BSDD	Faster R-CNN	80/10/10	10	16	0.2710	0.065	0.110	0.4709	1.0000	0.7331	0.5602	68.25
			50	8	1.2667	0.014	0.016	0.5780	0.8610	0.7170	0.5780	68.60
	Faster R-CNN	70/20/10	10	8	0.2875	0.027	0.027	0.2542	0.9701	0.6109	0.4767	68.42
			50	16	1.1167	0.011	0.011	0.4827	0.8678	0.6731	0.5215	68.05
	Faster R-CNN	70/10/20	10	16	0.2875	0.055	0.096	0.3480	0.9720	0.6580	0.5170	68.57
			50	8	1.1500	0.017	0.021	0.5530	**0.8690**	0.7090	0.5430	69.25

Note: Bold values in the recall column indicate the highest performance achieved for each model per dataset.

**Table 7:** Validation results from the YOLOv8 nano teacher model, used in the KD ablation study on the BDDCE dataset.

Classes	Precision	Recall	mAP@50	mAP@50-95
all	0.897	0.881	0.887	0.72
black spots	0.773	0.609	0.619	0.264
color variation	0.985	0.988	0.995	0.973
bottle	0.997	1	0.995	0.965
deformed thread	0.717	0.698	0.727	0.273
oval neck	0.941	1	0.995	0.897
no defect	0.967	0.993	0.993	0.944

**Table 8:** Performance of the teacher model on old classes during initial incremental learning stages.

Train			Test				
Time (h)	Box Loss	Class Loss	Class	Precision	Recall	mAP@50	mAP@50-95
0.580	0.429	0.287	all	0.932	0.910	0.913	0.745
			black spots	0.806	0.739	0.748	0.302
			color variation	0.991	0.991	0.995	0.966
			bottle	0.998	1	0.995	0.968

**Table 9:** Performance of the student model on all classes during final incremental learning stages.

Train					Test				
Time (h)	Student Loss	Teacher Loss	Class Loss	Box Loss	Class	Precision	Recall	mAP@50	mAP@50-95
2.247	1.189	2.020	0.007	0.908	all	0.778	0.836	0.809	0.617
					black spots	0.722	0.467	0.503	0.155
					color variation	0.827	1	0.990	0.901
					bottle	0.969	0.996	0.992	0.918
					deformed thread	0.636	0.676	0.599	0.198
					oval neck	0.876	0.919	0.937	0.778
					no defect	0.640	0.957	0.836	0.753

**Table 10:** Training results of the teacher model, using the BDDCE dataset with full class set (50 images per class).

Train			Test				
Time (min)	Box Loss	Class Loss	Class	Precision	Recall	mAP@50	mAP@50-95
7.5	0.400	0.379	all	0.858	0.873	0.880	0.686
			black spots	0.638	0.573	0.576	0.197
			color variation	0.953	1	0.988	0.952
			bottle	0.981	1	0.993	0.952
			deformed thread	0.746	0.775	0.772	0.275
			oval neck	0.929	1	0.995	0.854
			no defect	0.901	0.889	0.954	0.887

**Table 11:** Impact of the $5\times 10^{-5}$ learning rate during the ablation study.

Cycle	Time (min)	Student Loss	Teacher Loss	Class Loss	Box Loss	Precision	Recall	mAP@50	mAP@50-95	Inference (ms)
0	3.360	1.2649	1.8762	0.1122	0.8059	0.671	0.642	0.632	0.517	2.7
5	2.940	0.9691	1.6530	0.0218	0.7704	0.756	0.724	0.729	0.575	2.6
10	4.200	0.9110	1.7382	0.0137	0.7631	0.819	0.762	0.778	0.594	2.7
15	2.460	0.8854	1.8447	0.0131	0.7641	0.731	0.795	0.780	0.612	2.6
20	3.240	0.9999	2.0175	0.0117	0.7649	0.790	0.813	0.809	0.636	2.6

**Table 12:** Comparison of recall and mAP@50 between the first and final cycles across experiments 1–10.

Experiment	Recall (%)		mAP@50 (%)	
	First, Cycle	Final Cycle	First, Cycle	Final Cycle
1	63.0	83.0	61.2	81.4
2	61.3	79.0	57.4	80.2
3	53.0	80.8	52.1	76.1
4	60.2	77.1	31.1	71.5
5	60.2	78.3	33.1	71.8
6	60.9	76.2	34.2	72.4
7	62.8	76.2	55.3	74.0
8	60.5	72.8	32.7	73.7
9	59.8	80.1	31.4	74.5
10	61.5	72.9	45.7	73.8

**Figure A1:**
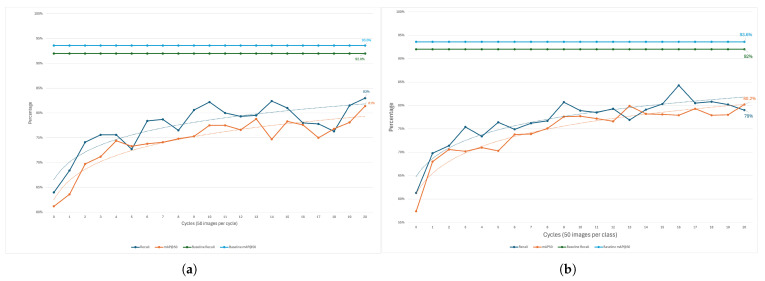

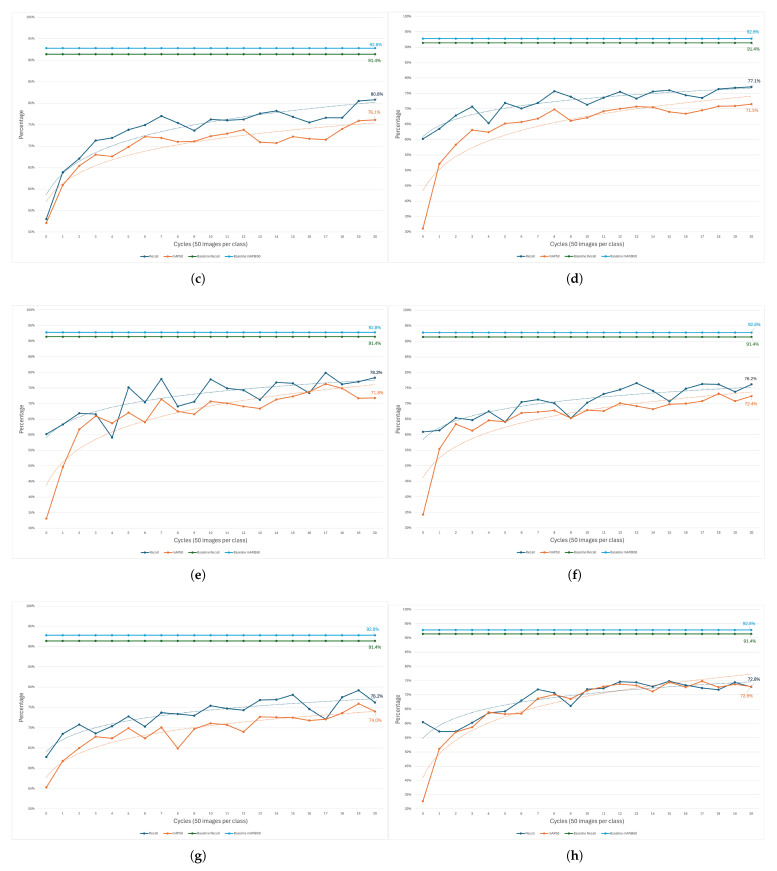

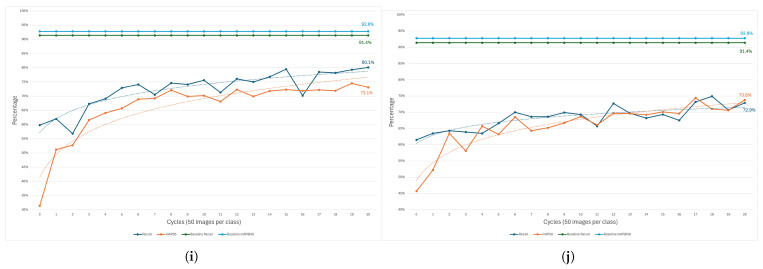
Evolution of recall and mAP@50 across 21 cycles for different datasets and setups: (**a**) BDDCE, full class set (50 images/class), (**b**) BDDCE, first 3 classes (50 images/class), (**c**) BDDIE, full class set (50 images/class), (**d**) BDDIE, first 3 classes (50 images/class), (**e**) BDDCE + BDDIE, full class set (50 images/class), (**f**) BDDCE + BDDIE, first 3 classes (50 images/class), (**g**) BDDCE, baseline, α=0.7, (**h**) BDDCE, baseline with first 3 classes, α=0.7, (**i**) BDDCE, baseline, α=0.2, (**j**) BDDCE, baseline with first 3 classes, α=0.2. Dotted lines represent a simple moving average to visualize the overall trend across cycles.

## Data Availability

The data presented in this study are available on request from the corresponding author. Data are not publicly available, as they constitute one of the major contributions from a publicly funded project.

## Abbreviations

The following abbreviations are used in this manuscript:

AI	Artificial Intelligence
BCE	Binary Cross Entropy
BDDCE	Bottle Defect Dataset in Controlled Environment
BDDIE	Bottle Defect Dataset in Industrial Environment
BSDD	Blade Surface Defect Dataset
CE	Cross Entropy
CIoU	Complete Intersection over Union
CNN	Convolutional Neural Network
CV	Computer Vision
CVAT	Computer Vision Annotation Tool
DFL	Distributed Focal Loss
DL	Deep Learning
EWC	Elastic Weight Consolidation
IoU	Intersection of Union
KD	Knowledge Distillation
LwF	Learning without Forgetting
mAP	Mean Average Precision
ML	Machine Learning
MSDD	Metal Surface Defect Dataset
NN	Neural Network
RCNN	Region-based Convolutional Neural Network
RILOD	Real-Time Incremental Learning for Object Detection
ROI	Region of Interest
SOA	State-of-the-Art
YOLO	You Only Look Once
